# Spontaneous retroperitoneal bleeding: a case series

**DOI:** 10.1186/1756-0500-7-659

**Published:** 2014-09-18

**Authors:** Hitoshi Yamamura, Takasei Morioka, Tomonori Yamamoto, Kazuhisa Kaneda, Yasumitsu Mizobata

**Affiliations:** Department of Critical Care Medicine, Graduate School of Medicine, Osaka City University, 1-4-3 Asahimachi, Abenoku, Osaka, 545-8585 Japan

**Keywords:** Spontaneous retroperitoneal bleeding, Nafamostat mesilate, Anticoagulant, Hemodialysis

## Abstract

**Background:**

We experienced four Japanese patients with spontaneous retroperitoneal bleeding, a rare disease. We categorized the clinical characteristics of spontaneous retroperitoneal bleeding in these patients treated in our hospital and discuss the risk factors of spontaneous retroperitoneal bleeding.

**Case presentation:**

Three of the 4 patients did not have a bleeding tendency as indicated by laboratory data obtained at the time of retroperitoneal bleeding. The causative blood vessels were the lumbar and superior gluteal arteries and the internal iliac artery. All patients were receiving an anticoagulant, heparin in one and nafamostat mesilate in the other three patients. Three patients were being treated with hemodialysis or continuous hemodiafiltration when the spontaneous retroperitoneal bleeding occurred. We achieved hemostasis with transcatheter arterial embolization in 3 patients and with surgical hemostasis in 1 patient.

**Conclusions:**

We suggest that in patients receiving anticoagulant therapy in whom progressive anemia and unstable vital signs are present, spontaneous retroperitoneal bleeding should be considered as a possible cause. Nafamostat mesilate may be one of the risk factors for spontaneous retroperitoneal bleeding.

## Background

Spontaneous retroperitoneal bleeding (SRB) is a rare disease. Most retroperitoneal bleeding is associated with trauma, tumor, hematologic disease, thrombotic thrombocytopenic purpura [1], or Evans syndrome [2]. Other than these existing diseases, heparin is one of the reported risk factors for SRB [3–6]. The clinical characteristics of SRB have not been elucidated yet. We experienced four patients with SRB in our intensive care units, of whom three patients were receiving nafamostat mesilate for anticoagulation. We categorize the clinical characteristics of SRB in these patients and discuss the risk factors for SRB.

## Case presentation

We experienced four Japanese patients with SRB in our intensive care and emergency care units. None of the patients had hematologic disease or congenital anomalies. We investigated the present and past medical histories, blood test results before bleeding, therapies used, and causative blood vessels of all four patients. SRB was diagnosed in all patients by computed tomography (CT) imaging, and no vessel malformations such as aneurysm, arteriovenous malformation, or arteriovenous shunt were found. We collected data relating to patient history, existing disease, dialysis, laboratory data, treatment, and prognosis. All patients gave their written informed consent for this data collection.

**Figure 1:**
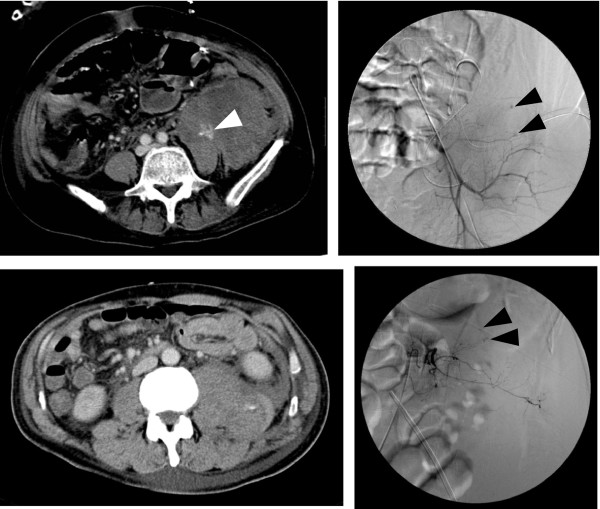
**Computed tomography and angiographic images in cases 1 and 2.** Upper left panel shows abdominal CT image from case 1. A giant intramuscular hematoma in the iliopsoas muscle and the active bleeding point (white arrowhead) are shown. Upper right panel shows an angiographic image from case 1. Extravasation was found in the superior gluteal artery area (black arrowheads). Lower left panel shows an abdominal CT image from case 2. A left iliopsoas muscle hematoma is shown. Lower right panel shows an angiographic image from case 2. Multiple extravasations were found in the 3rd lumbar artery (black arrowheads).

**Figure 2:**
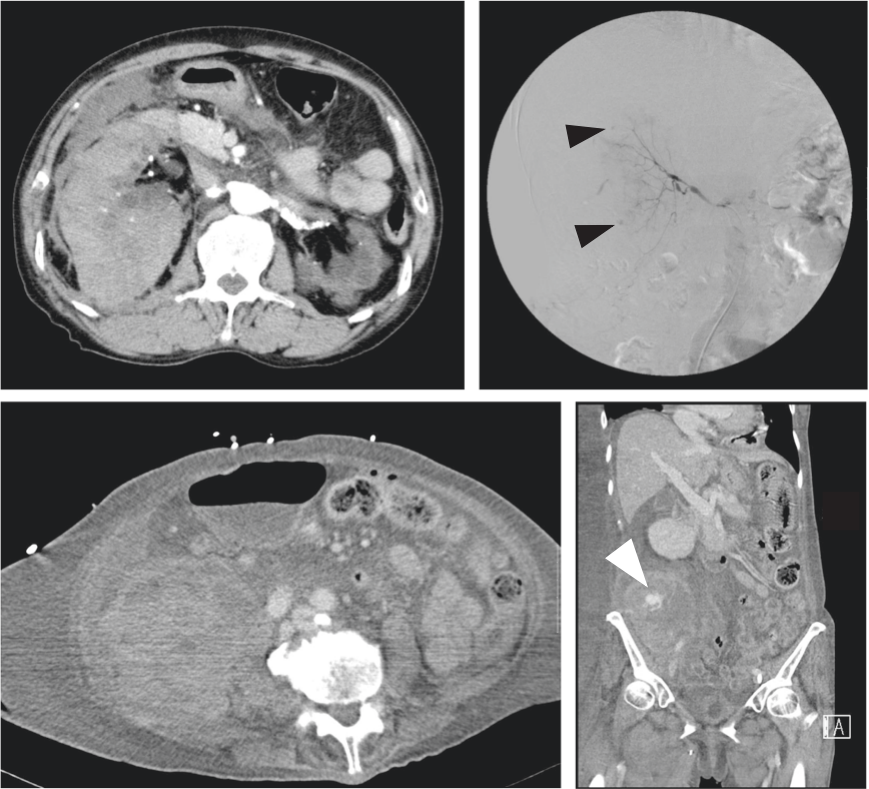
**Computed tomography and angiographic images in cases 3 and 4.** Upper left panel shows an abdominal CT image from case 3. A giant intramuscular hematoma in the right iliopsoas muscle is shown. Upper right panel shows an angiographic image from case 3. Extravasation was found in the 4th lumbar artery (black arrowheads). Lower panels show abdominal CT images from case 4 (left: axial view, right: coronal view). A giant retroperitoneal hematoma and the active bleeding point (white arrowhead) are shown.

Patient characteristics are listed in Table 1. The patients included 2 men and 2 women (average age, 63 years). Average platelet count before bleeding was 135,750/mm^3^ (range 113,000–175,000/mm^3^), prothrombin time (international normalized ratio) was 1.44 (range 1.03–1.84), and the activated partial thromboplastin time was 34.2 sec (range 30.5–39.0 sec). Only one of the three patients showed a bleeding tendency. The causative blood vessels were the lumbar and superior gluteal arteries and internal iliac artery. CT images showed that three patients had hematoma of the intra-iliopsoas muscle, and one patient had hematoma of the retroperitoneal space (Figures 1 and 2). None of the abdominal CT images showed the collection of any intraperitoneal fluid. We did not find aneurysm to be the cause of bleeding in any of the patients. Bleeding points in all patients were located in the distal portion of the causative artery on angiography, and we also observed micro-extravasation. We achieved hemostasis in 3 patients with transcatheter arterial embolization using SPONGEL® (Astellas, Tokyo, Japan) and in 1 patient with surgical hemostasis. We had begun hemodialysis before bleeding in two patients, whose durations of continuous hemodiafiltration had been 3 to 8 days, respectively, and one patient received hemodialysis every 3 days. Nafamostat mesilate was used for anticoagulation in all three of these patients. Three of the four patients were still alive at 1 month after the SRB incident, but the patient with fulminant hepatitis died from multiple organ failure on hospital day 19.

**Table 1:** **Patient characteristics**

Case	1	2	3	4
Age (yrs)	45	63	77	66
Sex	Male	Male	Female	Female
Diagnosis	Post liver transplantation	Fulminant hepatitis	Post cardiac arrest syndrome	Aneurysm of external iliac artery
Existing disease	Liver cirrhosis	(-)	Thyroid carcinoma	CRF, DM, HT
Bleeding artery	SGA, LA (3)	LA (3)	LA (1–4)	IIA
Hemodialysis	CHDF	CHDF	(-)	HD
Anticoagulant	NM	NM	Heparin	NM
PLT (×10^4^/mm^3^)	17.5	11.6	11.3	13.9
PT (INR)	1.48	1.84	1.03	1.42
APTT (sec)	30.5	39.0	30.6	36.6
Treatment	TAE	TAE	TAE	Operation
Duration in supine position (days)	13	12	10	2
Outcome	Survival	Death	Survival	Survival

*CRF*: Chronic renal failure, *DM*: Diabetes mellitus, *HT*: Hypertension, *SGA*: Superior gluteal artery, *LA*: Lumbar artery, *IIA*: Internal iliac artery, *CHDF*: Continuous hemodiafiltration, *HD*: Hemodialysis, *NM*: Nafamostat mesilate, *PLT*: Platelets, *PT (INR)*: Prothrombin time (international normalized ratio), *APTT*: Activated partial thromboplastin time, *TAE*: Transcatheter arterial embolization.

## Discussion

There are several well-recognized causes of retroperitoneal hematoma, including ruptured aortic aneurysm, traumatic vascular injury, retroperitoneal neoplasms, and coagulopathy [1]. However, idiopathic or spontaneous retroperitoneal hematoma is rare, and there are only a few documented reports implicating heparin, warfarin, low-molecular-weight heparin, or antiplatelet agents as a potential cause [3]. As a lesson from our cases, we consider administration of an anticoagulant drug and hemodialysis to be risk factors for SRB. There are no reports of nafamostat mesilate as a risk factor for SRB, to our knowledge, and we believe this is the first report.

Nafamostat mesilate is a serine protease inhibitor used clinically as an anti-inflammatory. It inhibits protein degrading enzymes such as thrombin, active form coagulation factors (XIIa, Xa, and VIIa), kallikrein, plasmin, and complements such as C1r, C1s, C3, and C5 convertases. It is also effective in inhibiting elements in the alternate pathway such as factors B and D. Similarly, it can counter the activation of key molecules in the coagulation cascade such as thrombin and plasmin and is thus used as an anticoagulant. The inhibitory action of thrombin develops without the need for antithrombin III. The half-life of nafamostat mesilate is short, 2–3 hours [7]. Use of nafamostat mesilate for anticoagulation is safe compared with that of heparin, and therefore, it is used in critical patients or those with a bleeding tendency.

We recognized hemorrhage in our patients on the basis of progressive anemia, tachycardia with low blood pressure, and abdominal distension. Abdominal ultrasonography was not useful for identifying retroperitoneal hemorrhage because none of our patients showed the presence of any intraabdominal fluid. However, enhanced CT was useful for the accurate diagnosis of SRB. Bleeding was slow in our patients, and thus it was hard to recognize SRB in the early phase. Trans-catheter embolization is reported to be useful in achieving hemostasis in SRB [8]. Multiple sites of bleeding were present in our patients, and bleeding occurred in peripheral arteries in three of the patients. SPONGEL® was used as the embolic material, and complete hemostasis was achieved in all patients with no incidents of re-bleeding or complications.

The mechanism of SRB was not clear. A previous report suggested that forceful muscular strain might be a mechanism of SRB [9]. In our patients and in previous reports, sites of bleeding were mostly intramuscular, occurring in the posterior region of the iliopsoas or gluteal muscles [9]. The sites of bleeding were the lumbar arteries in the majority of our patients. We considered one possible mechanism of blood vessel rupture in our patients to be the long time in which they had lain in the supine position, which resulted in compression of the posterior side of the involved muscle. Another possible mechanism might have been muscle strain of the iliopsoas muscle occurring during routine patient care of which neither the nursing staff nor the patients were aware. The patients with iliopsoas hematoma were in the supine position for an average of 11 days (range, 10–13 days), and changing of patient position was limited because they were being treated with continuous hemodiafiltration. Multiple sites of bleeding were present in all but one of our patients. Although our results may suggest that achievement of hemostasis in SRB with transcatheter arterial embolization was more useful than that by surgical treatment, this report included a very small number of cases from a single center. Thus, there is a need to collect more cases from additional centers. Further, we could not definitively determine that the cause of SRB was directly related to nafamostat mesilate. However, we suggest that nafamostat mesilate may be one risk factor for SRB.

## Conclusions

We suggest that SRB should be considered as a possible complication in patients receiving anticoagulant therapy in whom progressive anemia and unstable vital signs are present. Nafamostat mesilate may be one risk factor for SRB.

## Consent

Written informed consent was obtained from three of the patients themselves and from the next of kin of the fourth patient who died for publication of this Case Report and any accompanying images. A copy of the written consent is available for review by the Editor-in-Chief of this journal.

## Abbreviations

SRB: Spontaneous retroperitoneal bleeding
CT: Computed tomography.
